# New Appliances and Things Medical

**Published:** 1903-03-14

**Authors:** 


### NEW APPLIANCES AND THINGS MEDICAL.

[We shall be glad to receive, at our Office, 28 & 29 Southampton Street, Strand, London, W.C., from the manufacturers, specimens of all new preparations and appliances which may be brought out from time to time.]

### NEW HOT-WATER POUCHES FOR FEEDING-BOTTLES.

(Arthur Harrop, 3 Sutton Road, Heaton Norris, Stockport.)

These new patent indiarubber pouches consist of double-layered walls, which are to be filled with hot water; the central cavity is for receiving the feeding-botle, and almost completely surrounds it with a hot-water chamber. In this way a feeding-bottle can be kept warm for many hours. The pouch may be used for many other purposes, as, for instance, to keep the hands warm. While admitting the advantage of keeping a feeding-bottle warm for a considerable period of time under certain circumstances, we cannot approve of this idea for routine practice. If food is incubated in this manner it soon becomes absolutely unfitted for infant consumption, owing to the rapid development of bacteria under conditions so congenial to their growth. If the food has been previously completely sterilised, a difficult and undesirable operation, no untoward consequences owing to the growth of bacteria need be feared. We imagine, therefore, that these new pouches have a somewhat limited field of usefulness.

